# Diabetes, Plasma Glucose, and Incidence of Fatty Liver, Cirrhosis, and Liver Cancer: A Prospective Study of 0.5 Million People

**DOI:** 10.1002/hep.30083

**Published:** 2018-10-05

**Authors:** Yuanjie Pang, Christiana Kartsonaki, Iain Turnbull, Yu Guo, Robert Clarke, Yiping Chen, Fiona Bragg, Ling Yang, Zheng Bian, Iona Y. Millwood, Juanzhi Hao, Xianyong Han, Yajing Zang, Junshi Chen, Liming Li, Michael V. Holmes, Zhengming Chen

**Affiliations:** ^1^ Clinical Trial Service Unit & Epidemiological Studies Unit, Nuffield Department of Population Health University of Oxford Oxford UK; ^2^ Medical Research Council Population Health Research Unit, Nuffield Department of Population Health University of Oxford Oxford UK; ^3^ Chinese Academy of Medical Sciences Beijing China; ^4^ Qingdao Cancer Hospital Qingdao China; ^5^ Yongqinglu Community Health Service Center Qingdao China; ^6^ Qingdao Center for Disease Prevention and Control Qingdao China; ^7^ National Center for Food Safety Risk Assessment Beijing China; ^8^ School of Public Health Peking University; ^9^ National Institute for Health Research Oxford Biomedical Research Centre Oxford University Hospital Oxford UK

## Abstract

The prevalence of diabetes is increasing rapidly in China. However, evidence is limited about its effects on chronic liver diseases and liver cancer. We examined the associations of diabetes with chronic liver diseases and liver cancer and of random plasma glucose (RPG) with these liver diseases among participants without diabetes in Chinese adults and the possible interaction by hepatitis B virus (HBV) infection. The prospective China Kadoorie Biobank recruited 512,891 adults. During 10 years of follow‐up, 2,568 liver cancer, 2,082 cirrhosis, 1,298 hospitalized nonalcoholic fatty liver disease (NAFLD), and 244 hospitalized alcoholic liver disease (ALD) cases were recorded among 503,993 participants without prior history of cancer or chronic liver diseases at baseline. Cox regression was used to estimate hazard ratios (HRs) for each disease by diabetes status (previously diagnosed or screen‐detected) and, among those without previously diagnosed diabetes, by levels of RPG. Overall 5.8% of participants had diabetes at baseline. Compared to those without diabetes, individuals with diabetes had adjusted HRs of 1.49 (95% confidence interval 1.30‐1.70) for liver cancer, 1.81 (1.57‐2.09) for cirrhosis, 1.76 (1.47‐2.16) for NAFLD, and 2.24 (1.42‐3.54) for ALD. The excess risks decreased but remained elevated in those with longer duration. Among those without previously diagnosed diabetes, RPG was positively associated with liver diseases, with adjusted HRs per 1 mmol/L higher RPG of 1.04 (1.03‐1.06) for liver cancer, 1.07 (1.05‐1.09) for cirrhosis, 1.07 (1.05‐1.10) for NAFLD, and 1.10 (1.05‐1.15) for ALD. These associations did not differ by HBV infection. *Conclusion:* In Chinese adults, diabetes and higher blood glucose levels among those without known diabetes are associated with higher risks of liver cancer and major chronic liver diseases.

Chronic liver disease, including chronic hepatitis B virus (HBV) infection, is a major cause of morbidity and mortality in China.^1^ It affects approximately 400 million people, including 93 million carriers of hepatitis B surface antigen (HBsAg).^1^ Worldwide about 50% of deaths from liver cancer and 15% from cirrhosis occur in China.^2^, ^3^ Although age‐standardized adult mortality rates from liver cancer and cirrhosis have been declining in China, due partly to the elimination of aflatoxin from foodstuffs and partly to the decline in HBV seroprevalence since the 1990s,^4^ the incidence of certain other chronic liver diseases, including nonalcoholic fatty liver disease (NAFLD), is rising and accounts for an increasing proportion of liver disease in China.^1^

Previous studies of mostly Western populations have reported that individuals with diabetes have higher risks of liver cancer^4^, ^5^, ^6^ and cirrhosis,^5^, ^7^, ^8^, ^9^, ^10^, ^11^ while East Asian studies have shown a higher risk of NAFLD among individuals with diabetes.^12^, ^13^, ^14^, ^15^ However, the magnitude of the associated risks for different types of liver diseases and in different populations has not been clearly established. Among individuals without diabetes, substantial uncertainty also remains as to whether blood glucose levels are associated with increased risks of liver cancer and liver diseases and, if so, whether there is any threshold below which the association no longer exists. Furthermore, most previous studies, particularly those in East Asia, are not able to account for the effects of HBV infection,^4^ which remains the most important risk factor for liver cancer and cirrhosis in Asia^16^, ^17^ and may modify the associations between diabetes and liver diseases.

In China, the prevalence of diabetes has increased significantly in recent decades, from 2.5% in the 1990s to 11.6% in 2013, with a high proportion of cases undetected.^18^ Furthermore, the increase in diabetes prevalence is so recent that its impact on risk of liver cancer and liver diseases, if any, may not yet have fully emerged. Despite this, there is little reliable prospective evidence available in China about the associations of diabetes and blood glucose with risks of liver cancer and major chronic liver diseases. Appropriate understanding of their associations can inform risk prediction and disease prevention and treatment. We report relevant findings from the large prospective China Kadoorie Biobank (CKB) study.

## Participants and Methods

### STUDY POPULATION

Details of the CKB design, survey methods, and population characteristics have been described elsewhere.^19^ Briefly, 512,891 participants (210,222 men and 302,669 women) aged 30‐79 years were recruited into the study from 10 geographically defined localities (5 urban and 5 rural) in China during 2004‐2008. The study areas were selected to provide diversity in risk exposure and disease patterns, while taking into account population stability, quality of mortality and morbidity registries, capacity, and long‐term commitment within the areas. Potentially eligible participants in each of 100‐150 administrative units (rural villages or urban residential committees) selected for the study within each region were identified through official residential records, and invitation letters (with study information leaflets) were delivered door to door by local community leaders or health workers, following extensive publicity campaigns. Approximately 1.8 million persons were originally selected for recruitment to get the sample of 0.5 million (estimated population response rate ∼30%). Prior international, national, and regional ethics approvals were obtained; and all participants provided written informed consent.

### DATA COLLECTION

At local study assessment clinics, participants completed an interviewer‐administered, laptop‐based questionnaire on sociodemographic characteristics, smoking, alcohol consumption, diet, physical activity, personal and family medical history, and current medication. A range of physical measurements were recorded by trained technicians, including height, weight, hip and waist circumferences, bioimpedance, lung function, blood pressure, and heart rate, using calibrated instruments with standard protocols.

A 10‐mL nonfasting (with the time since the participant last ate recorded) blood sample was collected from participants into an ethylene diamine tetraacetic acid vacutainer (BD Hemogard; BD, USA). Immediate on‐site testing of the random plasma glucose (RPG) level was undertaken using the SureStep Plus System (Johnson & Johnson), regularly calibrated with the manufacturer's quality control solution. Participants with glucose levels ≥7.8 mmol/L and <11.1 mmol/L were invited to return for a fasting plasma glucose (FPG) test the next day. In addition, blood spot tests were used to measure HBsAg (ACON Biotech). RPG data were unavailable for 8,160 participants (because of a delay in making the on‐site test available in certain regions).

Previously diagnosed diabetes was defined by the question “Has a doctor ever told you that you had diabetes?”. Among positive respondents, additional information about age at diagnosis and current use of certain medications for the treatment of diabetes (e.g., insulin and metformin) and cardiovascular diseases (e.g., aspirin, lipid‐lowering, and blood pressure–lowering agents) was collected. Among those without previously diagnosed diabetes, screen‐detected diabetes was defined as (1) RPG ≥7.0 mmol/L if the time since last eating was ≥8 hours; (2) RPG ≥11.1 mmol/L if the time since last eating was <8 hours; or (3) FPG ≥7.0 mmol/L on subsequent testing.^20^ Of the 504,731 participants who provided blood samples, 108,200 fasted for at least 8 hours. Of 26,444 participants who did not fast and had an elevated RPG, 13,009 returned for an FPG test. For screen‐detected diabetes, 4,413 cases were based on RPG among individuals who fasted for ≥8 hours, 8,122 cases on RPG among individuals who fasted for <8 hours, and 1,582 cases on FPG on repeat testing.

From August to October 2008 (∼2.6 years after the baseline survey) 19,788 (∼5%) surviving participants were randomly selected to attend a resurvey. The data collection and survey procedures were much the same as in the baseline survey. RPG data were available for 19,712 (99.6%) resurvey participants.

### FOLLOW‐UP FOR MORBIDITY AND MORTALITY

The vital status of each participant was determined periodically through the Chinese National Center for Disease Control and Prevention's Disease Surveillance Points system and national health insurance system,^21^ supplemented by regular checks against local residential and administrative records and by annual active confirmation through street committees or village administrators. Additional information about major diseases and any episodes of hospitalization was collected through linkages, using each participant's unique national identification number, with disease registries (for cancer, ischemic heart disease, stroke, and diabetes) and national health insurance claims databases (for liver diseases), which has almost universal coverage in the study areas. All events were coded using the *International Classification of Diseases*, 10th revision (ICD‐10), by trained staff who were blinded to baseline information^19^ and reviewed centrally for consistency. The present study included incident liver diseases and liver cancer from enrollment until January 1, 2017 (a median of 10 years), by which time a total of 42,921 (8%) participants had died and 5,276 (1%) were lost to follow‐up. The classification and distribution of liver diseases by data source are shown in Supporting Table S1 (alcoholic liver disease [ALD] and NAFLD 100% by medical records). All analyses were restricted to first events of any liver diseases or liver cancer occurring between ages 35 and 79 years, with censoring when participants had died of nonliver diseases or were lost to follow‐up.

### STATISTICAL ANALYSIS

We excluded individuals with a prior history of cancer (n = 2,577) or cirrhosis or hepatitis at baseline (n = 6,321), leaving 503,993 individuals for the main analysis (Supporting Fig. S1).

Mean values and prevalence of baseline characteristics were calculated for diabetes status at baseline, standardized by 5‐year age groups, sex, and area structure of the CKB population.

Cox proportional hazards models were used to estimate adjusted hazard ratios (HRs) of specific disease incidence associated with diabetes and RPG levels, stratified by sex, study area (10 areas), and HBsAg, and adjusted for age at baseline, education (four groups: no formal school, primary school, middle/high school, or college/university), smoking (four groups: never regular, occasional, former regular, or current regular), alcohol (five groups: abstainers, ex‐weekly drinkers, reduced‐intake drinkers, occasional drinkers, or weekly drinkers), and total physical activity. Age was used as the underlying time scale. Cox models with a time‐updated explanatory variable for diabetes duration were used to estimate the association of diabetes duration with risk of liver diseases, with the same adjustment. *Duration* was defined as the time interval between diabetes diagnosis and time at risk. Among participants without diabetes at baseline (previously diagnosed or screen‐detected), incident diabetes cases (ICD‐10 code E11, n = 16,431) that occurred during the follow‐up were also included. We used Cox models with a time‐updated explanatory variable for diabetes, counting individuals with incident diabetes as exposed from their time of diagnosis. The analysis for incident diabetes also adjusted for prevalent diabetes. The analysis for RPG was conducted in participants without previously diagnosed diabetes and available RPG data (n = 480,304). RPG was categorized into four groups, ≤5.5 (reference), 5.6‐6.7, 6.8‐7.7, and ≥7.8 mmol/L, selected to include the FPG thresholds for impaired fasting glucose and diabetes. RPG was also modeled as a continuous variable to estimate risk associated with a 1 mmol/L higher level of RPG. The analysis for RPG was additionally adjusted for fasting time. For analyses involving more than two categories, all HRs are presented with “floating” standard errors to facilitate comparisons between groups.^22^

In sensitivity analyses, we further adjusted for diabetes medication (i.e., metformin and insulin) and comedication (i.e., statin and aspirin). In addition, as the HBsAg test only measured seroprevalence at baseline, we classified cirrhosis using both the baseline HBsAg results and diagnosis of viral hepatitis during follow‐up. *Viral cirrhosis* was defined as cirrhosis in individuals who had either a positive HBsAg test at baseline or a diagnosis of viral hepatitis (B15‐19) before cirrhosis, while the rest of the patients with cirrhosis were classified as having unknown cirrhosis. The analyses were conducted using SAS, version 9.3, and R, version 2.14.2.

## Results

### BASELINE CHARACTERISTICS AND INCIDENCE OF LIVER DISEASES

Among the 503,993 participants included, the mean (standard deviation [SD]) age was 51.5 (10.7) years and 59% were women. The prevalence of previously diagnosed and screen‐detected diabetes was 3.1% and 2.7%, respectively. The mean (SD) RPG was 5.7 (1.1), 11.8 (5.7), and 13.3 (5.4) mmol/L among participants without any diabetes, with previously diagnosed diabetes, and with screen‐detected diabetes, respectively. Individuals with diabetes were older and, after adjustment for age, sex, and region, more likely to have higher levels of body mass index (BMI), higher systolic blood pressure, lower physical activity, cardiovascular disease, hypertension, and a family history of diabetes (Table 1). Among those with previously diagnosed diabetes, the median age at first diagnosis was 53 years, and the median duration since diagnosis was 6.5 years. The median (interquartile range) of follow‐up was 10.1 (9.2, 11.1) years. During 10 years of follow‐up, there were 2,568 cases of liver cancer, 2,082 cases of cirrhosis, 1,298 cases of hospitalized NAFLD, and 244 cases of hospitalized ALD.

**Table 1:** Baseline Characteristics by Diabetes Status in CKB

		Diabetes Status		
	No Diabetes	Total	Previously Diagnosed	Screen‐Detected
Variable^a^	(n = 474,378)	(n = 29,615)	(n = 15,779)	(n = 13,836)
Age (SD), year	51.1 (10.6)	57.2 (9.6)	58.4 (9.1)	55.9 (9.9)
Female, %	59.0	61.7	62.3	61.0
Socioeconomic and lifestyle factors				
Urban resident, %	43.0	60.9	65.1	55.9
≥6 years of education, %	43.4	43.2	44.7	42.2
Household income ≥35,000 RMB/year, %	24.7	24.3	24.5	24.1
Ever regular smoking, %				
Male	67.8	66.2	64.4	67.6
Female	2.8	3.1	3.1	3.0
Weekly drinking, %				
Male	33.8	29.6	21.8	36.2
Female	2.1	1.3	0.7	1.9
Total physical activity (SD), MET hours/day	21.3 (13.9)	18.8 (11.9)	17.5 (10.6)	19.8 (12.9)
Anthropometry and blood pressure				
Height (SD), cm	158.6 (8.3)	158.8 (8.4)	159.0 (8.3)	158.5 (8.4)
BMI (SD), kg/m^2^	23.6 (3.3)	24.9 (3.6)	24.6 (3.5)	25.1 (3.7)
Waist circumference (SD), cm	80.0 (9.6)	84.9 (10.0)	84.3 (9.8)	85.3 (10.3)
Hip circumference (SD), cm	90.9 (6.8)	92.2 (7.6)	91.8 (7.5)	92.6 (7.7)
Waist to hip ratio (SD)	0.88 (0.07)	0.92 (0.07)	0.92 (0.07)	0.92 (0.07)
Percent body fat (SD)	27.9 (8.3)	30.4 (8.7)	29.4 (8.5)	31.1 (8.8)
BMI at age 25 (SD), kg/m^2^	21.9 (2.5)	22.8 (3.1)	23.2 (3.2)	22.5 (2.9)
SBP (SD), mm Hg	130.7 (21.0)	138.6 (22.6)	137.6 (22.6)	139.3 (22.7)
RPG (SD), mmol/L	5.7 (1.1)	12.6 (5.6)	11.8 (5.7)	13.3 (5.4)
HBsAg‐positive, %	2.7	2.9	2.7	3.1
Prior disease history, %				
CHD	2.8	5.2	6.9	3.2
Stroke or TIA	1.6	3.2	3.9	2.2
Hypertension	10.8	22.5	27.8	17.4
Family history of diabetes, %	4.5	12.2	16.1	9.2
Family history of cancer, %	13.9	14.1	15.1	13.3

a Results were adjusted for age, region, and sex (where appropriate). 1 mmol/L = 18 mg/dL.

Abbreviations: CHD, coronary heart disease; MET, metabolic equivalent of task; RMB, renminbi; SBP, systolic blood pressure; TIA, transient ischaemic attack.

### DIABETES AND RISKS OF LIVER DISEASES

Diabetes was associated with higher risks of liver diseases, with adjusted HRs of 1.49 (95% confidence interval 1.30‐1.70) for liver cancer, 1.81 (1.57‐2.09) for cirrhosis, 1.76 (1.47‐2.16) for NAFLD, and 2.24 (1.42‐3.54) for ALD (Table 2). The HRs were similar for previously diagnosed and screen‐detected diabetes (Supporting Table S2). Additional adjustment for BMI attenuated the association for NAFLD (HR, 1.33 [1.10‐1.62]) but had little effect on the associations with liver cancer, cirrhosis, and ALD (Table 2). The associations for these liver diseases persisted after excluding cases that occurred during the first 2 or 5 years of follow‐up (Supporting Table S3). For both liver cancer and cirrhosis, the HR appeared somewhat stronger for mortality than for incidence (liver cancer, 1.56 [1.34‐1.82] versus 1.49 [1.30‐1.70], *P* for heterogeneity 0.48; cirrhosis, 2.34 [1.65‐3.31] versus 1.81 [1.57‐2.09], *P* for heterogeneity 0.25; Supporting Table S4). When individuals with incident diabetes after baseline were also considered as exposed from their time of diagnosis, the associations were similar for liver cancer (1.62 [1.34‐1.95]), cirrhosis (1.78 [1.45‐2.18]), and ALD (2.03 [1.11‐3.73]), with a somewhat stronger association for NAFLD (3.39 [2.75‐4.18]). Compared to participants without diabetes, the risks of liver cancer, cirrhosis, and NAFLD were highest during 0 to <2 years following diabetes diagnosis, with HRs of 1.96 (1.45‐2.64), 2.62 (1.95‐3.52), and 5.13 (3.99‐6.58), respectively (Table 3). The excess risks decreased with increasing duration of diabetes but remained elevated even ≥10 years after diabetes diagnosis (HR, 1.42 [1.15‐1.75], 1.54 [1.21‐1.96], and 1.71 [1.31‐2.23]).

**Table 2:** Adjusted HRs for Liver Cancer and Chronic Liver Diseases by Diabetes Status

	No. Events	Rate, per 100,000	Model 1 HR (95% CI)	Model 2 HR (95% CI)
Liver cancer				
No diabetes	2,313	487.6	Reference	Reference
Diabetes	255	862.1	1.49 (1.30‐1.70)	1.52 (1.33‐1.74)
Cirrhosis				
No diabetes	1,858	397.4	Reference	Reference
Diabetes	224	756.4	1.81 (1.57‐2.09)	1.83 (1.59‐2.12)
Hospitalized NAFLD				
No diabetes	1,179	255.1	Reference	Reference
Diabetes	119	405.2	1.76 (1.47‐2.16)	1.33 (1.10‐1.62)
Hospitalized ALD				
No diabetes	223	52.5	Reference	Reference
Diabetes	21	81.0	2.24 (1.42‐3.54)	2.56 (1.61‐4.06)

Model 1: stratified by sex, region, and HBsAg status and adjusted for age at baseline, education, smoking, alcohol, and total physical activity.

Model 2: model 1 further adjusted for BMI.

Number of participants: no diabetes, n = 474,378; diabetes, n = 29,615.

Abbreviation: CI, confidence interval.

**Table 3:** Adjusted HRs for Liver Cancer and Chronic Liver Diseases by Duration of Diabetes^a^

	Liver Cancer		Cirrhosis		Hospitalized NAFLD		Hospitalised ALD	
	No. Events	HR (95% CI)	No. Events	HR (95% CI)	No. Events	HR (95% CI)	No. Events	HR (95% CI)
Incident diabetes	219	1.62 (1.34‐1.95)	216	1.78 (1.45‐2.18)	401	3.39 (2.75‐4.18)	25	2.03 (1.11‐3.73)
Diabetes								
No diabetes	2,259	1.00 (0.95‐1.05)	1,804	1.00 (0.95‐1.05)	1,075	1.00 (0.94‐1.07)	225	1.00 (0.86‐1.16)
0 to <2 years	65	1.96 (1.45‐2.64)	56	2.62 (1.95‐3.52)	84	5.13 (3.99‐6.58)	6	3.39 (1.53‐7.54)
2 to <5 years	96	1.54 (1.21‐1.96)	96	2.13 (1.69‐2.69)	64	2.10 (1.57‐2.81)	7	2.48 (1.24‐4.96)
5 to <10 years	110	1.42 (1.18‐1.72)	98	1.76 (1.45‐2.15)	56	2.10 (1.57‐2.23)	6	3.38 (2.07‐5.53)
≥10 years	37	1.42 (1.15‐1.75)	28	1.54 (1.21‐1.96)	19	1.71 (1.31‐2.23)	0	—
*P* for trend		0.10		0.003		<0.001		0.40

a Estimates were stratified by sex, region, and HBsAg status and adjusted for age at baseline, education, smoking, alcohol, and total physical activity. The HR for incident diabetes was also adjusted for prevalent diabetes. *P* for trend was calculated among participants with diabetes. For previously diagnosed diabetes and incident diabetes, duration was calculated as the time interval between age at diabetes diagnosis and age at risk. For screen‐detected diabetes, duration was calculated as the time interval between age at baseline and age at risk. Duration of diabetes was missing in 20 participants. Number of participants: no diabetes, n = 459,645; 0 to <2 years, n = 2,374; 2 to <5 years, n = 5,199; 5 to <10 years, n = 9,534; ≥10 years, n = 27,221.

### PLASMA GLUCOSE AND RISK OF LIVER DISEASES

**Figure 1:**
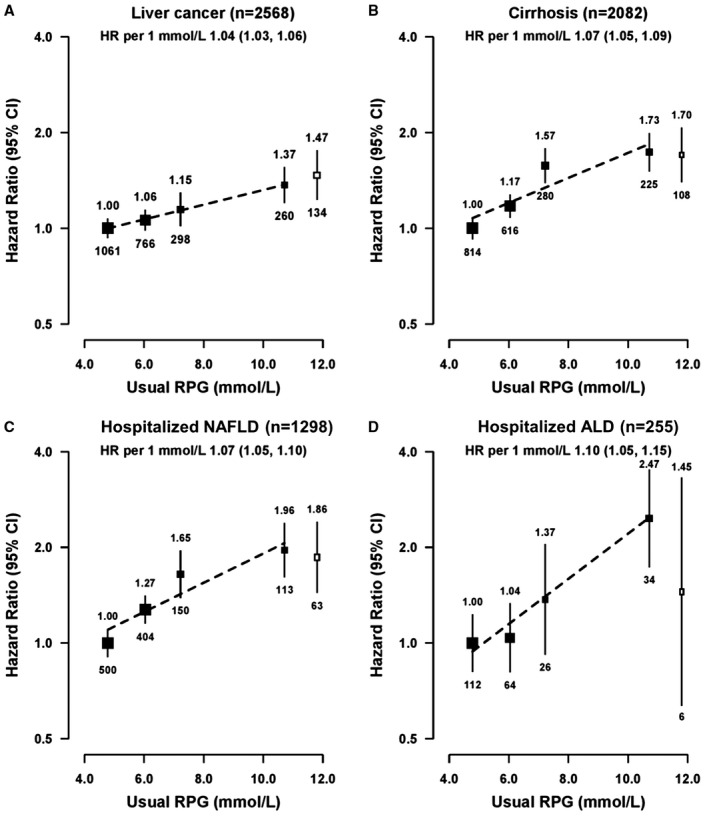
Adjusted HRs for liver cancer (A) and chronic liver diseases (B, C, D) by levels of RPG. Black boxes represent the HRs for RPG among participants without previously diagnosed diabetes, and the open box represents previously diagnosed diabetes. RPG levels for participants without previously diagnosed diabetes at baseline were classified as ≤5.5 (reference), 5.6‐6.7, 6.8‐7.7, and ≥7.8 mmol/L. HRs were plotted against the mean RPG level in each group. The size of the points is proportional to the inverse of the variance of the log HRs. Analyses were stratified by sex, region, and HBsAg and adjusted for age at baseline, education, smoking, alcohol, total physical activity, and fasting time. 1 mmol/L = 18 mg/dL. Number of participants: ≤5.5, n = 239,518; 5.6‐6.7, n = 156,602; 6.8‐7.7, n = 50,537; ≥7.8 mmol/L, n = 33,647. Abbreviation: CI, confidence interval.

In participants without previously diagnosed diabetes, there was a positive, and apparently log‐linear, association of RPG with risk of liver cancer (Fig. 1; Supporting Table S5), with each 1 mmol/L higher baseline RPG associated with an adjusted HR of 1.04 (1.03‐1.06). Stronger positive associations were found with cirrhosis (HR, 1.07 [1.05‐1.09] per 1 mmol/L higher RPG) and NAFLD (HR, 1.07 [1.05‐1.10]). Similarly, there was also a strong positive association with ALD (HR, 1.10 [1.05‐1.15]), although the number of events involved was small. As for diabetes, additional adjustment for BMI attenuated the association for NAFLD (HR, 1.04 [1.01‐1.06] per 1 mmol/L higher RPG) but had little effect on the associations with other liver diseases (Supporting Table S5).

### SUBGROUP ANALYSES AND SENSITIVITY ANALYSES

For both diabetes and RPG, the HRs for each specific disease were similar in HBsAg‐negative and HBsAg‐positive participants (Supporting Figs. S2 and S3). Likewise, the associations of plasma glucose with risk of liver diseases did not differ by fasting time (Supporting Fig. S3). For NAFLD, the HR for diabetes appeared greater in men than in women (2.42 [1.81‐3.23] versus 1.36 [1.05‐1.76]) and at younger ages (2.10 [1.31‐3.35] at 35‐49 years, 1.42 [1.00‐2.02] at 50‐59 years, and 1.58 [1.21‐2.05] at 60‐79 years) but did not differ significantly by other baseline characteristics. For cirrhosis and liver cancer, the associations with diabetes and RPG were similar in all population subgroups examined (Supporting Figs. S2 and S3). For ALD, the number of events was too small to permit such detailed subgroup analyses.

In sensitivity analyses, the associations of diabetes with risk of liver cancer and liver diseases did not change when further adjusting for use of diabetes medication or statin and aspirin (Supporting Table S6), except that the association with NAFLD became weaker when additionally adjusting for statin and aspirin use. Compared to participants without diabetes, the HRs for liver cancer, cirrhosis, and NAFLD appeared to be weaker among diabetes patients on metformin than for those not on any medication (Supporting Table S7). When assessing the associations with viral and unknown cirrhosis separately, similar associations were observed of diabetes and of RPG among participants without previously diagnosed diabetes (*P* for heterogeneity 0.79 and 0.99, respectively; Supporting Table S8).

## Discussion

We present a large prospective study of the associations of diabetes and blood glucose with risk of liver cancer and major chronic liver diseases in China. We have shown that individuals with diabetes have significantly higher risks of liver cancer, cirrhosis, and hospitalized NAFLD and ALD. The risks of liver cancer, cirrhosis, and NAFLD appeared to decrease with increasing duration of diabetes but remained elevated even 10 years after diabetes diagnosis. Among those without a prior diagnosis of diabetes, there were also strong positive associations of RPG with incidence of each of these diseases. Moreover, we showed that the associations for liver cancer and cirrhosis did not differ by HBV infection status.

Our finding linking diabetes with liver cancer was broadly consistent with those of previous prospective studies in Western and other East Asian populations.^4^ However, the magnitude of the risk estimate was somewhat lower than those described in two large collaborative pooled analyses of prospective studies (i.e., Emerging Risk Factors Collaboration and Asia‐Pacific Cohort Studies Collaboration).^5^, ^23^ These studies used liver cancer mortality as the endpoint, did not control for viral hepatitis, and involved smaller numbers of cases (533 and 93 deaths, respectively, versus 1,817 deaths in CKB). Our risk estimate for liver cancer incidence was generally comparable to those shown in prospective cohort studies in Korea (800 cases; HR, 1.54)^24^ and China (344 cases; HR, 1.64),^25^ as well as in a large record linkage study in Taiwan (19,207 cases; HR, 1.78),^26^ where the prevalence of HBV was comparable to that in our study. On the other hand, two prospective studies and three record linkage studies reported that diabetes was associated with 2‐fold to 3‐fold higher risk of cirrhosis.^5^, ^7^, ^8^, ^9^, ^10^, ^11^ Nearly all of these studies examined cirrhosis mortality,^7^, ^8^, ^10^, ^11^ and their risk estimates were stronger than in the present study of cirrhosis incidence. When we restricted the analysis to cirrhosis mortality, the risk estimate in CKB (HR, 2.34) was similar to those in the previous studies (HR, 2.0‐2.8). Furthermore, an Italian record linkage study (1,183 cirrhosis cases, including 260 ALD) reported an adjusted HR of 2.25 for ALD,^11^ which was consistent with our estimate in CKB.

In East Asia, HBV is the most important risk factor for liver cancer and cirrhosis.^16^ However, evidence has been inconclusive whether the associations of diabetes with liver cancer and cirrhosis differ by HBV infection. Three previous prospective studies reported a lower risk of liver cancer associated with diabetes in HBsAg‐positive than HBsAg‐negative participants,^4^ while two studies did not report any difference by HBsAg status.^4^ For cirrhosis, a study in Singaporean Chinese^10^ (133 cases) and an Italian study^11^ (389 cases) reported a stronger association for nonviral than viral cirrhosis classified using ICD‐10 codes. Moreover, each of these studies included fewer than 500 cases, so the power to detect any difference was limited. Our study involved >2,500 liver cancer and >2,000 cirrhosis cases and showed little difference in risk estimates by HBsAg status. However, the sensitivity of the HBsAg test in CKB was relatively low, and participants may have developed HBV infection during follow‐up, which may have affected the risk estimate.

Although a few prospective studies in East Asia have examined the associations of diabetes and plasma glucose with risk of NAFLD, their results have been inconclusive.^13^, ^14^, ^15^, ^27^ These studies involved occupational cohorts or those attending health checkup programs and diagnosed NAFLD cases with ultrasound but had short duration of follow‐up (median follow‐up 3‐5 years). Our estimate for hospital‐diagnosed NAFLD in CKB was comparable to two prospective cohort studies in China which involved 3,913 and 5,770 ultrasound‐detected NAFLD cases and reported ∼60% higher risk of NAFLD associated with diabetes.^12^, ^13^ Moreover, we showed that the positive association attenuated with increasing duration of diabetes but persisted even 10 years after diabetes diagnosis.

In addition to diabetes, at least 10 prospective cohort studies have reported on the relationship between blood glucose and liver cancer.^28^, ^29^ Most of these studies included fewer than 200 liver cancer cases, but in general they showed a positive association between blood glucose and liver cancer, regardless of how it was measured (random or fasting blood glucose). However, none of these previous studies excluded participants with diagnosed diabetes whose blood glucose levels might be controlled by medications, potentially diluting the association between blood glucose and liver cancer. On the other hand, we know of no studies investigating the association of blood glucose with cirrhosis risk, while prospective evidence with NAFLD risk has been inconclusive. In the CKB, we identified positive, dose–response associations of blood glucose with cirrhosis and NAFLD in participants without diabetes. More importantly, our findings also suggest that the positive associations of blood glucose with risks of liver cancer and other liver diseases continued down to below the diabetic and prediabetic ranges.

There are several possible mechanisms that might explain the associations of diabetes with liver diseases. On the one hand, the elevated risks of liver diseases following a diagnosis of diabetes may suggest surveillance bias. Indeed, several prospective cohort studies that used a time‐dependent approach reported 2‐fold to 4‐fold higher risks of liver cancer during the first 2 years following diabetes diagnosis,^30^, ^31^ which were consistent with our findings in CKB. However, these previous studies as well as our study showed that the excess risk of liver cancer remained even after 10 years following diabetes diagnosis, suggesting that diabetes might be an etiological factor for liver cancer and cirrhosis. On the other hand, hyperglycemia and insulin resistance might explain the link between diabetes and liver diseases.^32^ Insulin has growth‐promoting and mitogenic effects on cancer cells and may indirectly impact carcinogenesis by increasing bioavailability of insulin‐like growth factor.^32^ Insulin resistance can also lead to increased release of multiple proinflammatory cytokines (e.g., tumor necrosis factor‐alpha, interleukin‐6, and leptin) and chronic hepatic inflammation, which are associated with higher risks of cirrhosis and liver cancer.^32^ Furthermore, diabetes and NAFLD may co‐occur in the development of metabolic syndrome and share similar pathophysiological mechanisms, including hyperinsulinemia, hepatic insulin resistance, and dyslipidemia.^33^ In addition to hyperglycemia and insulin resistance, diet, microbiome, and obstructive sleep apnea may also explain the link between diabetes and liver diseases.^34^, ^35^, ^36^, ^37^

The strengths of CKB include a prospective design, a large and diverse study population, and careful adjustment for risk factors for liver diseases. In particular, we ascertained liver diseases through linkage to hospital records in addition to death and cancer registries, which allowed us to examine different types of liver diseases. To help assess possible reverse causality bias (i.e., diabetes induced by liver disease), we further examined the associations after excluding the first 2 or 5 years of follow‐up and conducted a time‐updated analysis.

This study also has limitations. First, our study relied on hospital records to capture NAFLD cases, which may have resulted in underdiagnosis of NAFLD. However, we ascertained all NAFLD cases diagnosed between 2013 and 2015 and showed that 93% of all hospitalized NAFLD cases were diagnosed by ultrasound or computed tomography. Moreover, the agreement of our risk estimates with previous studies of ultrasound‐detected NAFLD and the consistency of our risk estimates across population subgroups suggest that our results for NAFLD are probably real (Supporting Figs. S2 and S3). Second, liver diseases that were asymptomatic may be underdiagnosed (e.g., early‐stage liver cancer, compensated cirrhosis), which would bias the associations of diabetes and RPG with diseases. However, we showed similar associations of diabetes and RPG with liver diseases and liver cancer when excluding the first 2 or 5 years of follow‐up (Supporting Table S3). Moreover, our estimates for the associations of diabetes with liver cancer and cirrhosis were consistent with previous prospective studies that also ascertained disease outcomes using linkages to death and disease registries and health records, while our estimates for NAFLD were consistent with previous prospective studies that ascertained NAFLD using ultrasound or computed tomography (Supporting Table S11). Third, the use of self‐report may underestimate prevalent liver diseases at baseline. However, we showed similar associations when excluding those who had positive HBsAg tests and were overweight or obese (Supporting Figs. S2 and S3), both of which may increase risks of liver diseases. Moreover, in a nested case–control study of ∼18,000 participants with blood biochemistry data, we showed similar associations of diabetes and RPG with liver diseases when excluding participants with elevated alanine aminotransferase (Supporting Table S9). Fourth, although the prevalence of HBsAg positivity in CKB is lower than the national estimates,^1^, ^29^ a previous study reported this same assay as having a sensitivity of 95% and a specificity of 97%,^38^ suggesting good validity. Furthermore, our HBsAg test results showed expected associations with genetic susceptibility to HBV infection in the human leukocyte antigen region (unpublished data). On the other hand, we observed similar associations for viral cirrhosis and unknown cirrhosis when incorporating diagnosis of viral hepatitis during follow‐up (Supporting Table S8). Fifth, cases hospitalized for liver disease or cardiometabolic disease may be more likely to be screened for presence of NAFLD, potentially introducing detection bias. However, we found similar associations of diabetes and RPG with incident diagnosis of NAFLD when censoring cases with comorbidities prior to NAFLD diagnosis (Supporting Table S10) or when excluding NAFLD cases in the first 2 or 5 years of follow‐up (Supporting Table S3). Sixth, we used RPG, which might have measurement error or other within‐person variation. However, the measurement error is likely to be random, and we showed that the associations of RPG with liver diseases did not differ by fasting time (Supporting Fig. S3). Furthermore, a previous study in CKB showed that RPG is a good predictor of risk of cardiovascular disease.^39^

In summary, among Chinese adults, diabetes was associated with higher risks of liver cancer and major chronic liver diseases. Moreover, among participants without prior diagnosis of diabetes, RPG was positively associated with risks of liver cancer and chronic liver diseases. The associations for diabetes and RPG did not seem to differ by HBsAg status. Given that the majority of diabetes cases are undiagnosed in China, early detection of diabetes could help identify a group of individuals at increased risk of chronic liver diseases and prevent possible progression to cirrhosis and liver cancer.

## Potential conflict of interest

Nothing to report.

**Abbreviations**
ALD: alcoholic liver disease
BMI: body mass index
CKB: China Kadoorie Biobank
FPG: fasting plasma glucose
HBsAg: hepatitis B surface antigen
HBV: hepatitis B virus
HR: hazard ratio
ICD‐10: International Classification of Diseases, 10th Revision
NAFLD: nonalcoholic fatty liver disease
RPG: random plasma glucose
SD: standard deviation
