# Supplementary material for: Diabetes, Plasma Glucose, and Incidence of Fatty Liver, Cirrhosis, and Liver Cancer: A Prospective Study of 0.5 Million People
Source: Hepatology. 2018 Oct 5;68(4):1308–18. doi: 10.1002/hep.30083 (PMC6220764; doi:10.1002/hep.30083)
Supplement: Supplementary file 1 [file HEP-68-1308-s001.docx]

**Supporting Information**

**Diabetes, plasma glucose and incidence of liver cancer and major chronic liver diseases in Chinese: a prospective study of 0.5 million people**

**Hepatology**

Yuanjie Pang^1^, ScM; Christiana Kartsonaki^1,2^, DPhil; Iain Turnbull^1^, MRCP; Yu Guo^3^, MSc; Robert Clarke^1^, FRCP; Yiping Chen^1,2^, DPhil; Fiona Bragg^1^, DPhil; Ling Yang^1,2^, PhD; Zheng Bian^3^, MSc; Iona Y Millwood^1,2^, DPhil; Juanzhi Hao^4^, BSc; Xianyong Han^5^, MSc; Yajing Zang^6^, BSc; Junshi Chen^7^, MD; Liming Li^3,8^, MD; Michael V Holmes^1,2,9^, PhD; Zhengming Chen^1^, DPhil

1. Clinical Trial Service Unit & Epidemiological Studies Unit (CTSU), Nuffield Department of Population Health, University of Oxford, Oxford, UK
2. Medical Research Council Population Health Research Unit (MRC PHRU), Nuffield Department of Population Health, University of Oxford, Oxford, UK
3. Chinese Academy of Medical Sciences, 9 Dongdan San Tiao, Beijing 100730, China
4. Qingdao Cancer Hospital, Qingdao 266042, China
5. Yongqinglu Community Health Service Center, Qingdao 266041, China
6. Qingdao Center for Disease Prevention and Control, Qingdao 266033, China
7. School of Public Health, Peking University, Beijing 100191, China
8. National Center for Food Safety Risk Assessment, 37 Guangqu Road, Beijing 100021, China
9. National Institute for Health Research Oxford Biomedical Research Centre, Oxford University Hospital, Old Road, Oxford OX3 7LE, UK

**Address for correspondence**

Dr Christiana Kartsonaki

CTSU, Big Data Institute Building

Old Road Campus

University of Oxford

Oxford, OX3 7LF, UK

Fax: 44-1865-743985

Email: christiana.kartsonaki@ndph.ox.ac.uk

**Table of Contents**

[Supporting Table S1. Classification and distribution of liver diseases by ICD-10 codes in CKB 3](#_Toc509933709)

[Supporting Table S2. Adjusted HRs for liver cancer and chronic liver diseases by diabetes status 4](#_Toc509933710)

[Supporting Table S3. Adjusted HRs for liver cancer and chronic liver diseases by diabetes status after excluding early years of follow-up 5](#_Toc509933711)

[Supporting Table S4. Adjusted HRs for cirrhosis and liver cancer mortality by diabetes and levels of RPG among individuals without previously diagnosed diabetes 6](#_Toc509933712)

[Supporting Table S5. Adjusted HRs for liver cancer and chronic liver diseases by levels of RPG among participants without previously diagnosed diabetes 7](#_Toc509933713)

[Supporting Table S6. Adjusted HRs for liver cancer and chronic liver diseases by diabetes status 8](#_Toc509933714)

[Supporting Table S7. Adjusted HRs for liver cancer and chronic liver diseases by diabetes medications 9](#_Toc509933715)

[Supporting Table S8. Adjusted HRs for viral and unknown cirrhosis by diabetes and RPG participants without previously diagnosed diabetes 10](#_Toc509933716)

[Supporting Table S9. Adjusted HRs for liver cancer and chronic liver diseases by baseline adiposity excluding participants with elevated ALT 11](#_Toc509933717)

[Supporting Table S10. Adjusted HRs for hospitalized NAFLD by diabetes and RPG when censoring cases with comorbidities prior to NAFLD diagnosis 12](#_Toc509933718)

[Supporting Table S11. Selected key characteristics of published prospective studies of diabetes and risks of liver diseases 13](#_Toc509933719)

[Supporting Figure S1. Flow chart of study population for diabetes and RPG analyses 15](#_Toc509933720)

[Supporting Figure S2. Adjusted HRs for liver cancer, cirrhosis and NAFLD associated with diabetes in population subgroups 16](#_Toc509933721)

[Supporting Figure S3. Adjusted HRs for liver cancer, cirrhosis and NAFLD associated with 1 mmol/L higher RPG among individuals without previously diagnosed diabetes in population subgroups 17](#_Toc509933722)

# Supporting Table S1. Classification and distribution of liver diseases by ICD-10 codes in CKB

| **Liver disease** | **ICD-10 code** | **No. of cases** | | | |
| --- | --- | --- | --- | --- | --- |
|  |  | **Total** | **Health insurance** | **Death registry** | **Disease registry** |
| Liver cancer | C22 | 2568 | 1178 | 1847 | 974 |
| Cirrhosis | K74 | 2082 | 1603 | 713 | 5 |
| NAFLD | K76.0 | 1298 | 1298 | 0 | 0 |
| Alcoholic liver diseases | K70 | 244 | 244 | 0 | 0 |

# Supporting Table S2. Adjusted HRs for liver cancer and chronic liver diseases by diabetes status

|  | **No. events** | **Rate,**  **Per 100,000** | **Model 1**  **HR (95% CI)** | **Model 2**  **HR (95% CI)** |
| --- | --- | --- | --- | --- |
| **Liver cancer** |  |  |  |  |
| No diabetes | 2313 | 487.6 | 1.00 (0.95, 1.05) | 1.00 (0.95, 1.05) |
| Previously diagnosed diabetes | 134 | 849.3 | 1.47 (1.24, 1.75) | 1.50 (1.26, 1.78) |
| Screen-detected diabetes | 121 | 874.6 | 1.53 (1.28, 1.83) | 1.57 (1.31, 1.87) |
|  |  |  |  |  |
| **Cirrhosis** |  |  |  |  |
| No diabetes | 1858 | 391.7 | 1.00 (0.95, 1.05) | 1.00 (0.95, 1.06) |
| Previously diagnosed diabetes | 108 | 684.5 | 1.70 (1.40, 2.05) | 1.71 (1.42, 2.07) |
| Screen-detected diabetes | 116 | 838.5 | 1.92 (1.60, 2.30) | 1.95 (1.62, 2.34) |
|  |  |  |  |  |
| **Hospitalized NAFLD** |  |  |  |  |
| No diabetes | 1179 | 255.1 | 1.00 (0.93, 1.07) | 1.00 (0.93, 1.07) |
| Previously diagnosed diabetes | 63 | 399.3 | 1.86 (1.45, 2.39) | 1.46 (1.14, 1.88) |
| Screen-detected diabetes | 56 | 412.0 | 1.73 (1.33, 2.24) | 1.24 (0.95, 1.60) |
|  |  |  |  |  |
| **Hospitalized ALD** |  |  |  |  |
| No diabetes | 223 | 52.5 | 1.00 (0.85, 1.17) | 1.00 (0.84, 1.18) |
| Previously diagnosed diabetes | 6 | 38.0 | 1.45 (0.65, 3.25) | 1.67 (0.74, 3.73) |
| Screen-detected diabetes | 15 | 130.1 | 2.80 (1.69, 4.65) | 3.18 (1.92, 5.27) |

Model 1: stratified by sex, region, and HBsAg, and adjusted for age at baseline, education, smoking, alcohol, and total physical activity.

Model 2: Model 1 plus BMI.

# Supporting Table S3. Adjusted HRs for liver cancer and chronic liver diseases by diabetes status after excluding early years of follow-up^1^

|  | **No. events** | **Rate,**  **Per 100,000** | **Excluding first 2**  **years of follow-up**  **HR (95% CI)** | **No. events** | **Rate,**  **Per 100,000** | **Excluding first 5 years of follow-up**  **HR (95% CI)** |
| --- | --- | --- | --- | --- | --- | --- |
| **Liver cancer** |  |  |  |  |  |  |
| No diabetes | 1904 | 405.2 | Reference | 1237 | 268.7 | Reference |
| Diabetes | 204 | 706.1 | 1.48 (1.28, 1.72) | 154 | 561.4 | 1.78 (1.50, 2.12) |
| RPG per 1 mmol/L^2^ | 1958 | 407.6 | 1.04 (1.02, 1.06) | 1275 | 265.4 | 1.05 (1.03, 1.07) |
|  |  |  |  |  |  |  |
| **Cirrhosis** |  |  |  |  |  |  |
| No diabetes | 1595 | 339.4 | Reference | 1081 | 234.8 | Reference |
| Diabetes | 181 | 626.4 | 1.75 (1.50, 2.05) | 118 | 430.1 | 1.76 (1.45, 2.14) |
| RPG per 1 mmol/L^2^ | 1654 | 344.3 | 1.07 (1.05, 1.08) | 1117 | 232.5 | 1.06 (1.03, 1.08) |
|  |  |  |  |  |  |  |
| **Hospitalized NAFLD** |  |  |  |  |  |  |
| No diabetes | 960 | 209.6 | Reference | 739 | 163.8 | Reference |
| Diabetes | 93 | 325.3 | 1.80 (1.48, 2.18) | 69 | 251.5 | 1.83 (1.51, 2.22) |
| RPG per 1 mmol/L^2^ | 931 | 199.1 | 1.07 (1.05, 1.10) | 716 | 153.1 | 1.07 (1.05, 1.10) |
|  |  |  |  |  |  |  |
| **Hospitalized ALD** | |  |  |  |  |  |
| No diabetes | 211 | 47.0 | Reference | 141 | 32.4 | Reference |
| Diabetes | 19 | 76.1 | 2.42 (1.53, 3.83) | 12 | 51.0 | 2.37 (1.42, 3.94) |
| RPG per 1 mmol/L^2^ | 212 | 47.5 | 1.10 (1.05, 1.15) | 142 | 31.8 | 1.10 (1.05, 1.16) |

^1^ Estimates were stratified by sex, region, and HBsAg, and adjusted for age at baseline, education, smoking, alcohol, and total physical activity.

^2^ HRs for RPG were done among participants without previously diagnosed diabetes and additionally adjusted for fasting time.

# Supporting Table S4. Adjusted HRs for cirrhosis and liver cancer mortality by diabetes and levels of RPG among individuals without previously diagnosed diabetes

|  | **No. events** | **Rate,**  **Per 100,000** | **Model 1**  **HR (95% CI)** | **Model 2**  **HR (95% CI)** |
| --- | --- | --- | --- | --- |
| **Liver cancer** |  |  |  |  |
| Diabetes |  |  |  |  |
| No | 1631 | 343.8 | Reference | Reference |
| Yes | 186 | 628.2 | 1.56 (1.34, 1.82) | 1.61 (1.38, 1.88) |
| RPG |  |  |  |  |
| ≤ 5.5 | 740 | 308.7 | 1.00 (0.93, 1.08) | 1.00 (0.93, 1.08) |
| 5.5 - 6.7 | 553 | 353.1 | 1.11 (1.02, 1.20) | 1.11 (1.03, 1.21) |
| 6.8 - 7.7 | 217 | 429.2 | 1.20 (1.05, 1.37) | 1.22 (1.06, 1.39) |
| ≥ 7.8 | 180 | 535.2 | 1.37 (1.18, 1.59) | 1.41 (1.21, 1.63) |
| ***Effects per 1 mmol/L*** | ***1690*** | ***311.5*** | 1.05 (1.03, 1.07) | 1.06 (1.04, 1.08) |
| *p for trend* |  |  | *<0.001* | *<0.001* |
|  |  |  |  |  |
| **Cirrhosis** |  |  |  |  |
| Diabetes |  |  |  |  |
| No | 213 | 44.9 | Reference | Reference |
| Yes | 40 | 135.1 | 2.34 (1.65, 3.31) | 2.37 (1.67, 3.36) |
| RPG |  |  |  |  |
| ≤ 5.5 | 76 | 31.7 | 1.00 (0.79, 1.27) | 1.00 (0.79, 1.27) |
| 5.5 - 6.7 | 70 | 44.7 | 1.16 (0.92, 1.47) | 1.17 (0.93, 1.47) |
| 6.8 - 7.7 | 41 | 81.1 | 1.76 (1.29, 2.39) | 1.77 (1.30, 2.42) |
| ≥ 7.8 | 41 | 121.9 | 2.43 (1.78, 3.33) | 2.47 (1.81, 3.39) |
| ***Effects per 1 mmol/L*** | ***228*** | ***41.2*** | 1.09 (1.04, 1.13) | 1.09 (1.05, 1.13) |
| *p for trend* |  |  | *<0.001* | *<0.001* |

Model 1: stratified by sex, region, and HBsAg, and adjusted for age at baseline, education, smoking, alcohol, total physical activity, and fasting time (for RPG).

Model 2: Model 1 plus BMI.

# Supporting Table S5. Adjusted HRs for liver cancer and chronic liver diseases by levels of RPG among participants without previously diagnosed diabetes

|  | **No. events** | **Rate,**  **Per 100,000** | **Model 1**  **HR (95% CI)** | **Model 2**  **HR (95% CI)** |
| --- | --- | --- | --- | --- |
| **Liver cancer** |  |  |  |  |
| ≤ 5.5 | 1061 | 442.9 | 1.00 (0.94, 1.07) | 1.00 (0.94, 1.07) |
| 5.5 - 6.7 | 766 | 489.1 | 1.06 (0.99, 1.13) | 1.07 (0.99, 1.14) |
| 6.8 - 7.7 | 298 | 589.7 | 1.15 (1.02, 1.28) | 1.16 (1.03, 1.30) |
| ≥ 7.8 | 260 | 772.7 | 1.37 (1.21, 1.55) | 1.40 (1.24, 1.59) |
| ***Effects per 1 mmol/L*** | ***2385*** | ***496.5*** | 1.04 (1.03, 1.06) | 1.05 (1.03, 1.06) |
| *p* for trend |  |  | *<0.001* | *<0.001* |
|  |  |  |  |  |
| **Cirrhosis** |  |  |  |  |
| ≤ 5.5 | 814 | 339.8 | 1.00 (0.93, 1.08) | 1.00 (0.93, 1.08) |
| 5.5 - 6.7 | 616 | 393.3 | 1.17 (1.09, 1.27) | 1.18 (1.09, 1.27) |
| 6.8 - 7.7 | 280 | 554.1 | 1.57 (1.40, 1.78) | 1.59 (1.41, 1.79) |
| ≥ 7.8 | 225 | 668.7 | 1.73 (1.52, 1.98) | 1.76 (1.54, 2.02) |
| ***Effects per 1 mmol/L*** | ***1935*** | ***402.8*** | 1.07 (1.05, 1.09) | 1.07 (1.05, 1.09) |
| *p* for trend |  |  | *<0.001* | *<0.001* |
|  |  |  |  |  |
| **Hospitalized NAFLD** |  |  |  |  |
| ≤ 5.5 | 500 | 215.4 | 1.00 (0.91, 1.10) | 1.00 (0.91, 1.10) |
| 5.5 - 6.7 | 404 | 263.7 | 1.27 (1.16, 1.40) | 1.17 (1.06, 1.28) |
| 6.8 - 7.7 | 150 | 308.7 | 1.65 (1.40, 1.94) | 1.39 (1.18, 1.63) |
| ≥ 7.8 | 113 | 338.8 | 1.96 (1.62, 2.36) | 1.44 (1.19, 1.74) |
| ***Effects per 1 mmol/L*** | ***1167*** | ***249.6*** | 1.07 (1.05, 1.10) | 1.04 (1.01, 1.06) |
| *p* for trend |  |  | *<0.001* | *<0.001* |
|  |  |  |  |  |
| **Hospitalized ALD** | |  |  |  |
| ≤ 5.5 | 112 | 49.7 | 1.00 (0.82, 1.21) | 1.00 (0.82, 1.22) |
| 5.5 - 6.7 | 64 | 44.7 | 1.04 (0.82, 1.33) | 1.07 (0.84, 1.36) |
| 6.8 - 7.7 | 26 | 55.4 | 1.37 (0.93, 2.02) | 1.43 (0.97, 2.11) |
| ≥ 7.8 | 34 | 110.0 | 2.47 (1.76, 3.50) | 2.66 (1.88, 3.75) |
| ***Effects* per 1 mmol/L** | **236** | **52.9** | 1.10 (1.05, 1.15) | 1.11 (1.06, 1.16) |
| *p* for trend |  |  | *<0.001* | *<0.001* |

Model 1: stratified by sex, region, and HBsAg, and adjusted for age at baseline, education, smoking, alcohol, total physical activity, and fasting time.

Model 2: Model 1 plus BMI.

# Supporting Table S6. Adjusted HRs for liver cancer and chronic liver diseases by diabetes status

|  | **No. events** | **Model 1**  **HR (95% CI)** | **Model 2**  **HR (95% CI)** | **Model 3**  **HR (95% CI)** |
| --- | --- | --- | --- | --- |
| **Liver cancer** |  |  |  |  |
| No diabetes | 2313 | Reference | Reference | Reference |
| Diabetes | 255 | 1.49 (1.30, 1.70) | 1.53 (1.28, 1.83) | 1.56 (1.36, 1.80) |
|  |  |  |  |  |
| **Cirrhosis** |  |  |  |  |
| No diabetes | 1858 | Reference | Reference | Reference |
| Diabetes | 224 | 1.81 (1.57, 2.09) | 1.92 (1.60, 2.30) | 1.87 (1.61, 2.18) |
|  |  |  |  |  |
| **Hospitalized NAFLD** |  |  |  |  |
| No diabetes | 1179 | Reference | Reference | Reference |
| Diabetes | 119 | 1.78 (1.47, 2.16) | 1.71 (1.32, 2.22) | 1.41 (1.15, 1.74) |
|  |  |  |  |  |
| **Hospitalized ALD** |  |  |  |  |
| No diabetes | 223 | Reference | Reference | Reference |
| Diabetes | 21 | 2.24 (1.42, 3.54) | 3.11 (1.96, 4.93) | 2.26 (1.40, 3.67) |

Model 1: stratified by sex, region, and HBsAg, and adjusted for age at baseline, education, smoking, alcohol, and total physical activity.

Model 2: Model 1 plus diabetes medication.

Model 3: Model 1 plus statin and aspirin.

# Supporting Table S7. Adjusted HRs for liver cancer and chronic liver diseases by diabetes medications^1^

|  | **No. events** | **HR (95% CI)** |
| --- | --- | --- |
| **Liver cancer** |  |  |
| No diabetes | 2313 | 1.00 (0.95, 1.06) |
| No medication | 56 | 1.73 (1.33, 2.25) |
| Metformin | 63 | 1.28 (1.00, 1.63) |
| Insulin | 12 | 1.63 (0.92, 2.87) |
| Metformin and insulin | 3 | 1.64 (0.53, 5.09) |
|  |  |  |
| **Cirrhosis** |  |  |
| No diabetes | 1858 | 1.00 (0.94, 1.07) |
| No medication | 48 | 2.15 (1.62, 2.85) |
| Metformin | 44 | 1.25 (0.93, 1.67) |
| Insulin | 14 | 2.66 (1.57, 4.52) |
| Metformin and insulin | 2 | 1.56 (0.39, 6.23) |
|  |  |  |
| **Hospitalized NAFLD** |  |  |
| No diabetes | 1179 | 1.00 (0.92, 1.09) |
| No medication | 26 | 2.05 (1.39, 3.00) |
| Metformin | 26 | 1.59 (1.08, 2.33) |
| Insulin | 7 | 1.61 (0.76, 3.39) |
| Metformin and insulin | 4 | 7.74 (2.90, 20.63) |
|  |  |  |
| **Hospitalized ALD** |  |  |
| No diabetes | 234 | 1.00 (0.83, 1.21) |
| No medication | 1 | 0.62 (0.09, 4.44) |
| Metformin | 3 | 1.41 (0.45, 4.36) |
| Insulin | 0 | – |
| Metformin and insulin | 2 | 20.94 (5.20, 84.33) |

^1^ Estimates were stratified by sex, region, and HBsAg, and adjusted for age at baseline, education, smoking, alcohol, and total physical activity.

Number of participants: no diabetes, n=474,378; no medication, n=5495; metformin, n=8362; insulin, n=1553; metformin and insulin, n=369. Participants with screen-detected diabetes were excluded from the analysis.

# Supporting Table S8. Adjusted HRs for viral and unknown cirrhosis by diabetes and RPG participants without previously diagnosed diabetes^1^

|  | **No. events** | **Rate,**  **Per 100,000** | **HR (95% CI)** |
| --- | --- | --- | --- |
| **Viral cirrhosis** |  |  |  |
| No diabetes | 681 | 143.6 | Reference |
| Diabetes | 79 | 266.8 | 1.82 (1.44, 2.32) |
| ≤ 5.5 | 311 | 129.8 | 1.00 (0.89, 1.12) |
| 5.5 - 6.7 | 227 | 145.0 | 1.19 (1.04, 1.35) |
| 6.8 - 7.7 | 100 | 197.9 | 1.48 (1.21, 1.80) |
| ≥ 7.8 | 86 | 255.6 | 1.68 (1.35, 2.08) |
| ***Effects per 1 mmol/L*** | ***724*** | ***150.7*** | 1.07 (1.04, 1.10) |
| *p* for trend |  |  | *<0.001* |
|  |  |  |  |
| **Unknown cirrhosis** |  |  |  |
| No diabetes | 1177 | 248.1 | Reference |
| Diabetes | 145 | 489.6 | 1.80 (1.51, 2.15) |
| ≤ 5.5 | 503 | 209.8 | 1.00 (0.91, 1.10) |
| 5.5 - 6.7 | 389 | 248.5 | 1.16 (1.05, 1.28) |
| 6.8 - 7.7 | 180 | 356.2 | 1.61 (1.39, 1.87) |
| ≥ 7.8 | 139 | 413.1 | 1.74 (1.47, 2.05) |
| ***Effects per 1 mmol/L*** | ***1211*** | ***252.1*** | 1.07 (1.04, 1.09) |
| *p* for trend |  |  | *<0.001* |

^1^ Estimates were stratified by sex, region, and HBsAg, and adjusted for age at baseline, education, smoking, alcohol, total physical activity, and fasting time.

*p* for heterogeneity: diabetes, 0.79; RPG, 0.99.

# Supporting Table S9. Adjusted HRs for liver cancer and chronic liver diseases by baseline adiposity excluding participants with elevated ALT^1^

|  | **Nested case-control** | **Exclusion by ALT^2^** |
| --- | --- | --- |
|  | **HR (95% CI)** | **HR (95% CI)** |
| **No. of participants** | 17 972 | 16 456 |
|  |  |  |
| **Liver cancer** |  |  |
| **No. of cases** | 97 | 86 |
| **Diabetes** | 1.03 (0.49, 2.18) | 0.83 (0.30, 2.33) |
| **RPG per 1 mmol/L** | 1.06 (1.00, 1.11) | 1.01 (0.92, 1.10) |
|  |  |  |
| **Cirrhosis** |  |  |
| **No. of cases** | 104 | 69 |
| **Diabetes** | 2.01 (1.12, 3.59) | 2.31 (1.02, 5.25) |
| **RPG per 1 mmol/L** | 1.06 (1.00, 1.12) | 1.04 (0.95, 1.13) |
|  |  |  |
| **Hospitalized NAFLD** |  |  |
| **No. of cases** | 51 | 46 |
| **Diabetes** | 1.16 (0.46, 2.96) | 1.14 (0.34, 3.79) |
| **RPG per 1 mmol/L** | 0.99 (0.89, 1.11) | 0.97 (0.83, 1.14) |

Abbreviations: ALT, alanine aminotransferase.

^1^ Estimates were stratified by sex, region, and HBsAg, and adjusted for age at baseline, education, smoking, alcohol, total physical activity, and case-control status in the nested case-control study.

^2^ Exclusion: baseline cancer, cirrhosis, hepatitis, HBsAg positive, heavy alcohol drinkers (men ≥60 g/d and ≥women 40 g/d), and ALT ≥40 IU/l.

# Supporting Table S10. Adjusted HRs for hospitalized NAFLD by diabetes and RPG when censoring cases with comorbidities prior to NAFLD diagnosis^1^

| **Comorbidities** | **No. of cases** | **Diabetes** | **RPG per 1 mmol/L** | | |
| --- | --- | --- | --- | --- | --- |
|  |  | **HR (95% CI)** | | **HR (95% CI)** |  |
| **Overall** | 1298 | 1.78 (1.47, 2.16) | | 1.07 (1.05, 1.10) |  |
| **Cardio-metabolic^2^** | 842 | 1.06 (0.78, 1.44) | | 1.05 (1.01, 1.08) |  |
| **Cardio-metabolic and others^3^** | 726 | 1.15 (0.84, 1.59) | | 1.05 (1.02, 1.09) |  |

^1^ Estimates were stratified by sex, region, and HBsAg, and adjusted for age at baseline, education, smoking, alcohol, and total physical activity.

^2^ Comorbidities included cardiovascular diseases (I00-I09, I16-I88, I95-I99, I10-I15 [only where fatal]), and diabetes (E10-E14).

^3^ Comorbidities included cardiovascular diseases (I00-I09, I16-I88, I95-I99, I10-I15 [only where fatal]), diabetes (E10-E14), respiratory diseases (J00-J99), and cancer (C00-C97).

# Supporting Table S11. Selected key characteristics of published prospective studies of diabetes and risks of liver diseases

| **Reference** | **Study population** | **No. of cases/median follow-up** | **Definition of diabetes** | **Liver disease ascertainment** | **Outcome** | **Results** |
| --- | --- | --- | --- | --- | --- | --- |
| *Liver cancer* |  |  |  |  |  |  |
| Campbell et al 2016^6^ | 1.6 M participants in the Liver Cancer Pooling Project in the US | 2162/10+ years | Self-reported and screen-detected diabetes | Death and cancer registries, health records | Incidence | HR 2.61 (2.34, 2.91) |
| Yang et al 2013^25^ | 133,288 participants in the Shanghai Men’s and Women’s Health Study in China | 344/10+ years | Self-reported | Death and cancer registries | Incidence | HR  M 1.63 (1.06, 2.51)  F 1.64 (1.03, 2.61) |
| Lo et al 2012^26^ | 1.8 M participants in the National Health Research Institute record linkage study in Taiwan | 19,207/3.5 years | Hospital diagnosis | Health records | Incidence | HR 1.78 (1.73, 1.84) |
| Emerging Risk Factor Collaboration 2011^5^ | 0.8 M participants in 97 prospective studies | 533/13.6 years | Self-reported and screen-detected diabetes | Death registry | Mortality | HR 2.16 (1.62, 2.88) |
| Lam et al 2010^23^ | 367,361 participants in 36 Asian and Australian cohort studies | 93/4 years | Self-reported and screen-detected diabetes | Death registry | Mortality | HR 1.51 (1.19, 1.91) |
| Jee et al 2005^24^ | 1.3 M participants in the Korean Cancer Prevention Study | 800/10 years | Self-reported and screen-detected diabetes | Death and cancer registries, health records | Incidence | HR  M 1.59 (1.45, 1.74)  F 1.28 (1.00, 1.66) |
| *Cirrhosis* |  |  |  |  |  |  |
| Goh et al 2016^10^ | 63,275 participants in the Singapore Chinese Health Study | 133/16.9 years | Self-reported | Death registry | Mortality | HR 2.80 (2.04, 3.83) |
| Zoppini et al 2014^11^ | 167,621 diabetic patients in a regional electronic archive in Italy | 1183/5+ years | Hospital diagnosis | Death registry | Mortality | SMR 2.17 (1.90, 2.47) |
| Davis et al 2012^7^ | 6450 participants in the Fremantle Diabetes Study in Australia | 167/11 years | Hospital diagnosis | Health records | Incidence | IRR 1.66 (1.37, 2.02) |
| Emerging Risk Factor Collaboration 2011^5^ | 0.8 M participants in 97 prospective studies | 1429/13.6 years | Self-reported and screen-detected diabetes | Death registry | Mortality | HR 2.28 (1.90, 2.74) |
| El-Serag et al 2004^9^ | 0.8 M patients discharged from Veterans Affairs Hospitals in the US | 2339/10+ years | Hospital diagnosis | Death registry and health records | Incidence | HR 1.98 (1.88, 2.09) |
| De Marco et al 1999^8^ | 7148 diabetes patients in Italy | 68/5 years | Hospital diagnosis | Death registry | Mortality | SMR 2.52 (1.96, 3.20) |
| *NAFLD* |  |  |  |  |  |  |
| Li et al 2017^12^ | 18,111 participants of retired employees in China | 2697/4.6 years | Self-reported and screen-detected diabetes | Ultrasound | Incidence | HR 1.64 (1.45, 1.86) |
| Zhang et al 2015^13^ | 15,791 participants who underwent a routine health check-up programme in China | 3913/3.3 years | Self-reported diabetes | Ultrasound | Incidence | HR 1.43 (1.10, 1.86) |
| Xu et al 2013^14^ | 6,403 participants who underwent a routine health check-up programme in China | 494/5 years | Fasting blood glucose | Ultrasound | Incidence | HR FBG per 1 mmol/L  0.89 (0.77, 1.04) |
| Sung et al 2012^15^ | 2589 participants who had an occupational health check-up in Korea | 430/4.4 years | Fasting blood glucose | Ultrasound | Incidence | HR FBG per 1 mmol/L  1.22 (1.04, 1.42) |

Abbreviations: HR, hazard ratio; IRR, incidence rate ratio; FBP, fasting blood glucose; SMR, standardised mortality ratio; M, male; F, female.

# Supporting Figure S1. Flow chart of study population for diabetes and RPG analyses


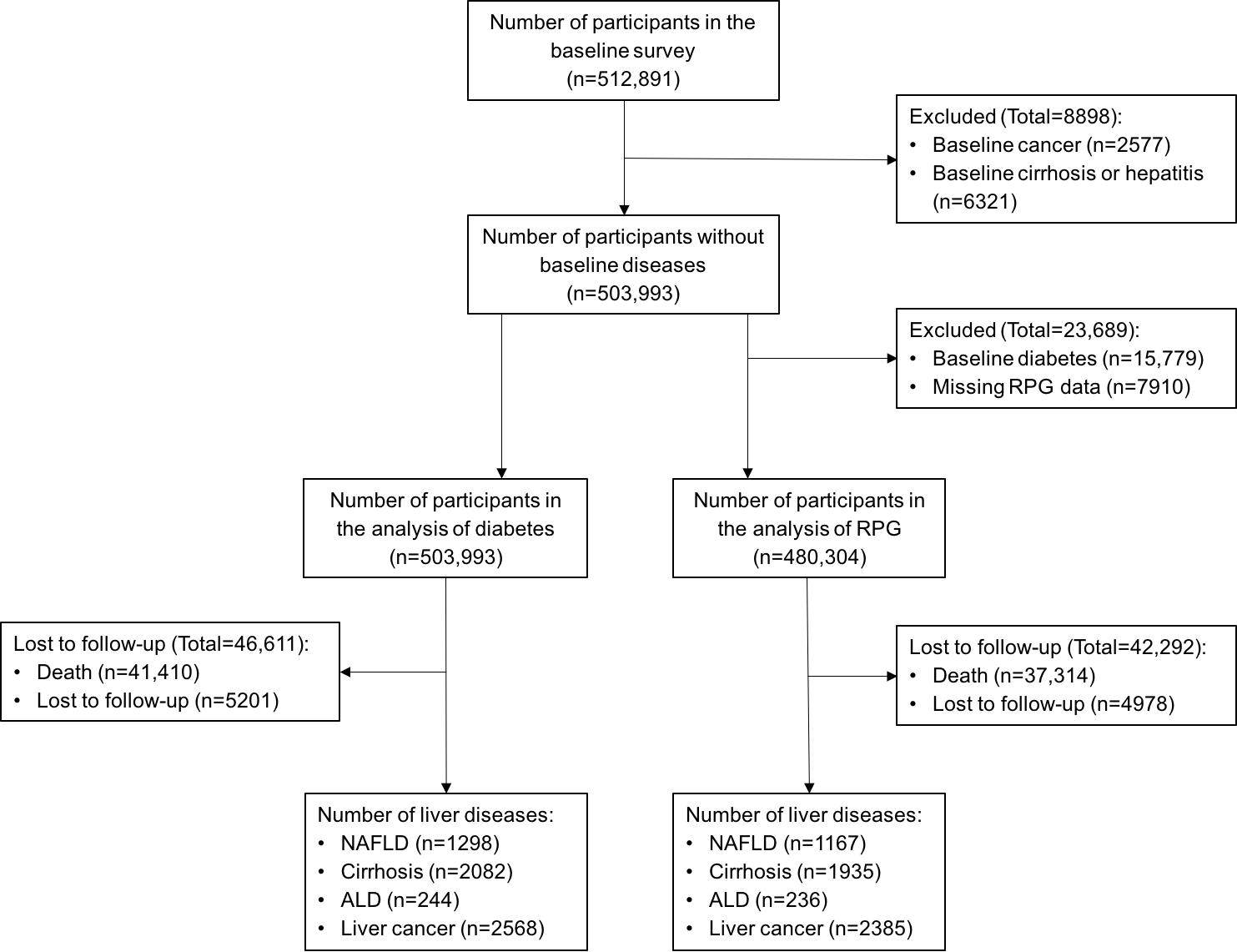


# Supporting Figure S2. Adjusted HRs for liver cancer, cirrhosis and NAFLD associated with diabetes in population subgroups

**
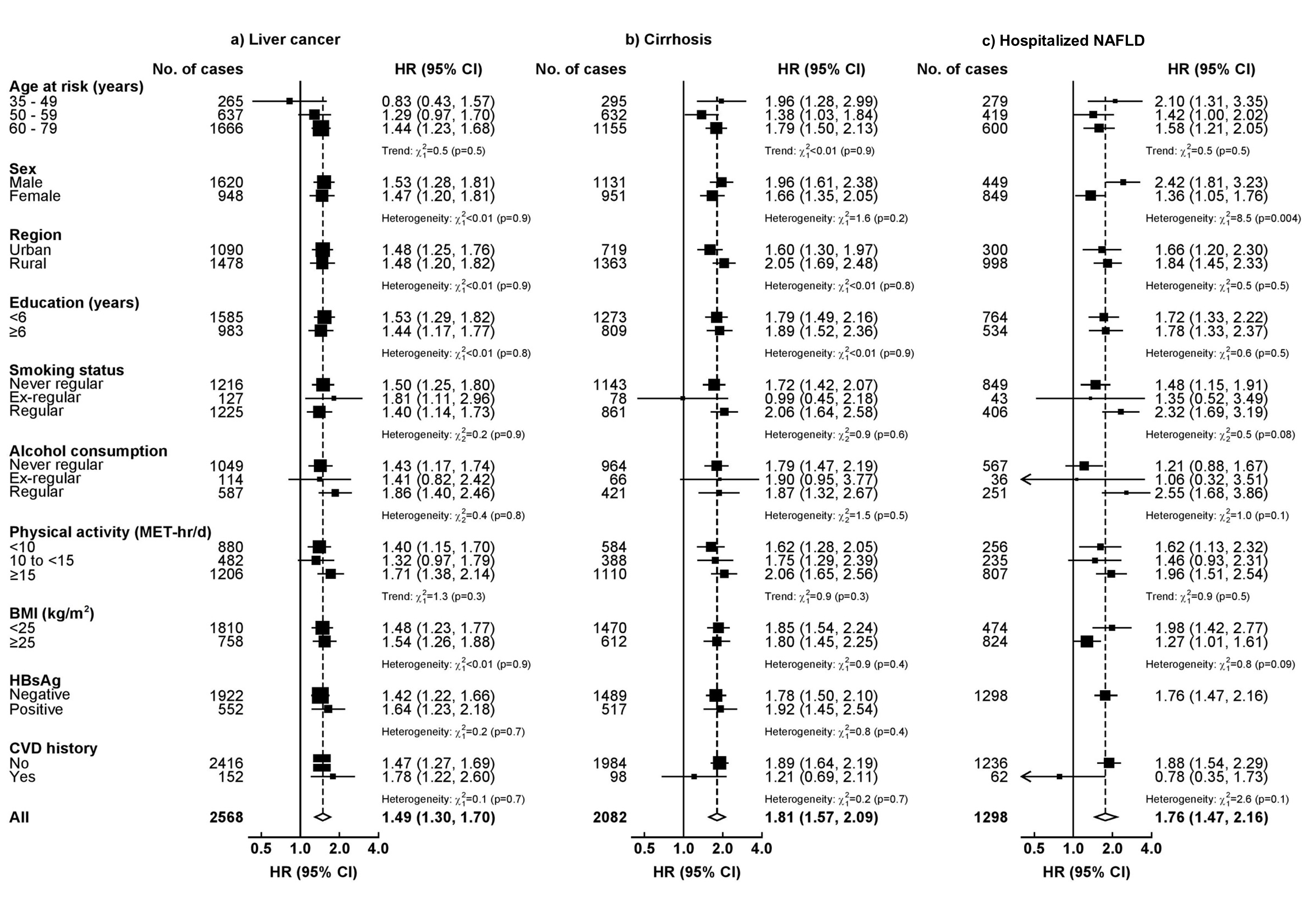
**

The analyses were stratified by sex, region, and HBsAg, and adjusted for age at baseline, education, smoking, alcohol, and total physical activity, where appropriate. Boxes represent subgroup-specific estimates and diamonds represent the overall HR. The sizes of the boxes are proportional to the inverse of the variance of the log hazard ratios.

# Supporting Figure S3. Adjusted HRs for liver cancer, cirrhosis and NAFLD associated with 1 mmol/L higher RPG among individuals without previously diagnosed diabetes in population subgroups


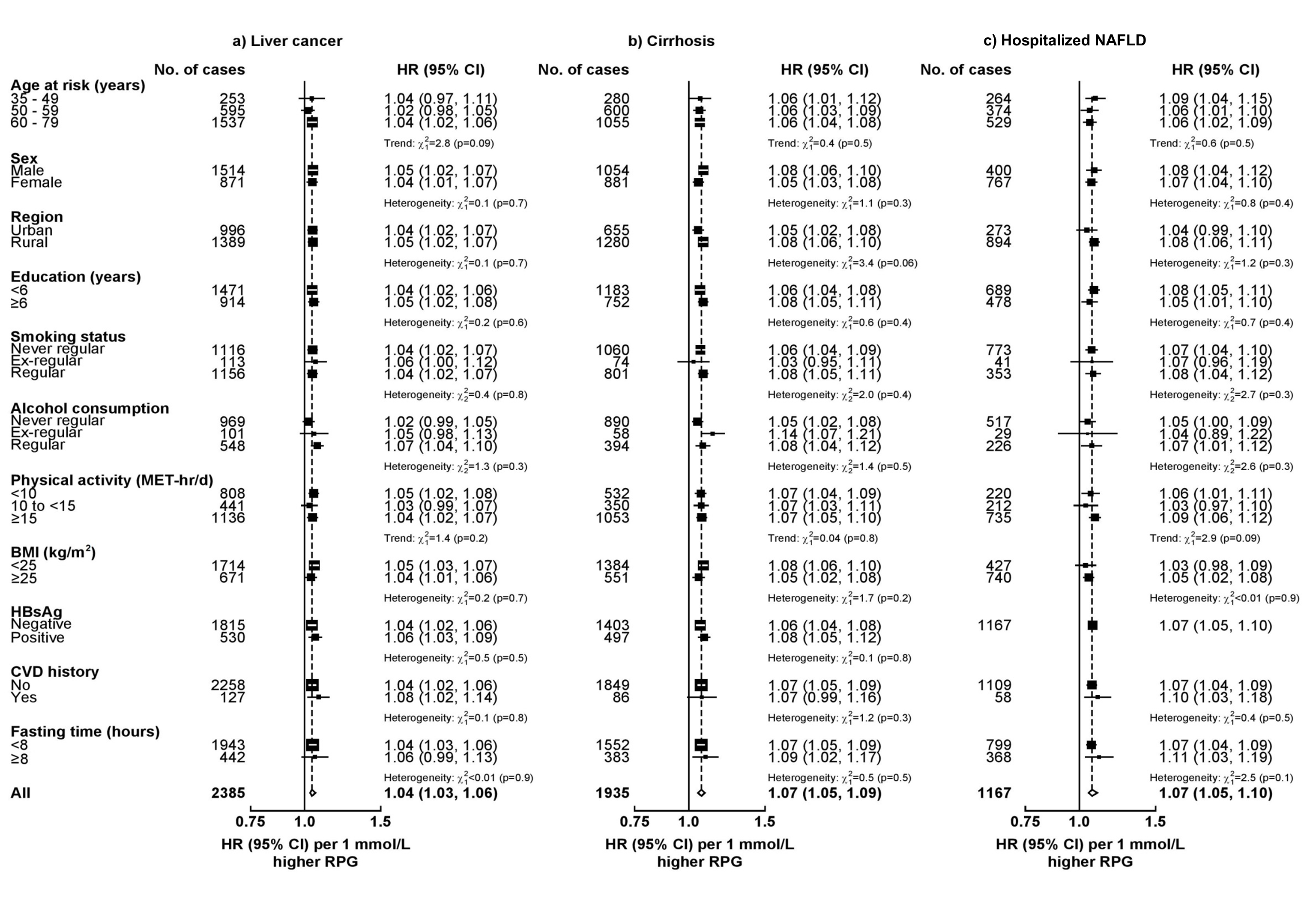


Convention as in Supporting Figure S2. 1 mmol/L= 18mg/dL.
